# Persistent *Salmonella enterica* serovar Typhimurium Infection Increases the Susceptibility of Mice to Develop Intestinal Inflammation

**DOI:** 10.3389/fimmu.2018.01166

**Published:** 2018-05-29

**Authors:** Bárbara M. Schultz, Geraldyne A. Salazar, Carolina A. Paduro, Catalina Pardo-Roa, Daniela P. Pizarro, Francisco J. Salazar-Echegarai, Javiera Torres, Claudia A. Riedel, Alexis M. Kalergis, Manuel M. Álvarez-Lobos, Susan M. Bueno

**Affiliations:** ^1^Millennium Institute on Immunology and Immunotherapy, Departamento de Genética Molecular y Microbiología, Facultad de Ciencias Biológicas, Pontificia Universidad Católica de Chile, Santiago, Chile; ^2^Departamento de Anatomía Patológica, Facultad de Medicina, Pontificia Universidad Católica de Chile, Santiago, Chile; ^3^Millennium Institute on Immunology and Immunotherapy, Departamento de Ciencias Biológicas, Facultad de Ciencias de la Vida, Universidad Andrés Bello, Santiago, Chile; ^4^Departamento de Endocrinología, Facultad de Medicina, Pontificia Universidad Católica de Chile, Santiago, Chile; ^5^Departamento de Gastroenterología, Facultad de Medicina, Pontificia Universidad Católica de Chile, Santiago, Chile

**Keywords:** *Salmonella enterica* serovar Typhimurium, inflammatory bowel disease, colitis, interleukin-10, persistence, dextran sulfate sodium

## Abstract

Chronic intestinal inflammations are triggered by genetic and environmental components. However, it remains unclear how specific changes in the microbiota, host immunity, or pathogen exposure could promote the onset and exacerbation of these diseases. Here, we evaluated whether *Salmonella enterica* serovar Typhimurium (*S*. Typhimurium) infection increases the susceptibility to develop intestinal inflammation in mice. Two mouse models were used to evaluate the impact of *S*. Typhimurium infection: the chemical induction of colitis by dextran sulfate sodium (DSS) and interleukin (IL)-10^−/−^ mice, which develop spontaneous intestinal inflammation. We observed that *S*. Typhimurium infection makes DSS-treated and IL-10^−/−^ mice more susceptible to develop intestinal inflammation. Importantly, this increased susceptibility is associated to the ability of *S*. Typhimurium to persist in liver and spleen of infected mice, which depends on the virulence proteins secreted by *Salmonella* Pathogenicity Island 2-encoded type three secretion system (TTSS-2). Although immunization with a live attenuated vaccine resulted in a moderate reduction of the IL-10^−/−^ mice susceptibility to develop intestinal inflammation due to previous *S*. Typhimurium infection, it did not prevent bacterial persistence. Our results suggest that persistent *S*. Typhimurium infection may increase the susceptibility of mice to develop inflammation in the intestine, which could be associated with virulence proteins secreted by TTSS-2.

## Introduction

Inflammatory bowel diseases (IBDs) are chronic intestinal immune disorders that include Crohn’s disease (CD) and ulcerative colitis (UC) (1). Both have increased their incidence worldwide in the last decades and are becoming an important social and economic burden (2, 3). Although the etiology of IBD remains unknown, the development of the disease requires a genetic component and an environmental trigger. There are multiple genetic factors associated with both diseases (110 and 163 genes are associated with UC and CD, respectively) (4). However, genetic components contribute only in a minor proportion to the development of IBD (5). On the other hand, the development of the disease requires one or more environmental factors that trigger the onset of disease, such as certain types of foods—such as standard western diet—(6), estrogens (7, 8), cigarette smoke (9), age (10), dysbiosis (10–12), and gastrointestinal infection by enteric bacterial pathogens (13–15). Considering a major role of the environmental factors, pathogenic bacteria infection not only may be an important factor that increase the intestinal inflammation but it could be also related to an early onset of the disease (16).

*Salmonella* is a genus of Gram negative, facultative intracellular and pathogenic bacteria able to infect mammals and other hosts (17). *Salmonella enterica* serovar Typhimurium (*S*. Typhimurium) is a common gastrointestinal pathogen and a public health problem worldwide, being the main cause of gastroenteritis and an important cause of zoonotic infection (18). This pathogen can survive and persist inside phagocytic cells, such as macrophages and dendritic cells, resulting in an asymptomatic but infectious carrier state that could be triggered by some antibiotic treatments (19, 20). *S*. Typhimurium has several virulence factors that prevent activation of the host immune response, favoring dissemination of the bacteria to other tissues (21, 22). Genes found in large chromosomal regions, known as *Salmonella* Pathogenicity Island 1 (SPI-1) and *Salmonella* Pathogenicity Island 2 (SPI-2), encode the most relevant virulence factors contributing to *S*. Typhimurium infection. Both pathogenicity islands encode type three secretion systems (TTSSs) (22, 23). The TTSS coded by genes in SPI-1 (TTSS-1) allows the bacterial entry into non-phagocytic cells, such as intestinal epithelial cells (24, 25) and promote inflammation in the gastrointestinal tract to alter the intestinal microbiota (21). The function of the TTSS coded by genes in the SPI-2 (TTSS-2) is important for intracellular infection of phagocytic cells, prevention of antigen presentation by dendritic cells and inhibition of T cell activation, to favor infection of deep organs such as the spleen and liver (21, 26–28). Moreover, strains lacking TTSS-2 are less virulent and are not able to cause systemic infection due to an impairment of intracellular survival and Peyer’s patches colonization (21, 23).

Several changes take place in the epithelial barrier after an infection with *S*. Typhimurium (29, 30), which probably also promote a permanent alteration of the gut immune response (28). For this reason, *S*. Typhimurium has been proposed as a potential environmental risk factor able to trigger intestinal inflammation in susceptible host. To address this hypothesis, we tested whether *S*. Typhimurium infection enhance intestinal inflammation in two murine models for IBD: mice treated with dextran sulfate sodium (DSS) and interleukin (IL)-10^−/−^ mice. In both models, we observed that mice previously infected with *S*. Typhimurium showed higher inflammation of the intestine, as compared to uninfected mice. Importantly, mice infected with virulent *S*. Typhimurium and treated with enrofloxacin still showed persistent bacteria in feces, spleen, and liver after 4 weeks of infection. Further, inflammation was associated to TTSS-2 expression, since infection with a strain lacking a functional TTSS-2 was unable to cause permanent inflammatory damage of the intestine. In summary, our results suggest that infection with *S*. Typhimurium increases the susceptibility of intestinal chronic inflammation in two mouse models that resemble IBD susceptible patients, due to the ability of the bacteria to cause persistent infection.

## Materials and Methods

### Mice

Six to eight-weeks-old female C57BL/6 wild-type (WT) mice and interleukin-10 knockout mice (IL-10^−/−^) were obtained from Jackson Laboratory (Bar Harbor, ME, USA) and maintained in the specific pathogen-free animal facility at the Facultad de Ciencias Biológicas, Pontificia Universidad Católica de Chile. This study was approved by the Scientific Ethical Committee for Animal and Environment Care of the Pontificia Universidad Católica de Chile and the Scientific Ethical Committee for Research Biosafety (Protocol number 160715006). Experiments were conducted in agreement to institutional and international Guidelines for Animal Care.

### Bacterial Strains and Growth Conditions

*Salmonella enterica* serovar Typhimurium 14028 was originally obtained from the America Type Culture Collection and kindly provided by Dr. Carlos Santiviago, Universidad de Chile, Santiago, Chile. The WT or mutants strains were stored at −80°C in Luria-Bertani (LB) medium supplemented with 20% glycerol. To prepare infection doses, a small aliquot of the frozen bacteria was inoculated in LB medium and grown with agitation at 37°C, overnight. Then, bacteria were diluted 1/100 in LB medium and grown with agitation at 37°C until OD_600 nm_ equal to 0.6 was reached. Mutant strains ΔTTSS-1 (*invC*) was generated by Eichelberg and colleagues by inserting a kanamycin resistance cassette by double homologous recombination (31). The ΔTTSS-2 and ΔSPI-2 were generated Tobar and colleagues (26) by replacing the *spiC* gene and the whole SPI-2 by a kanamycin cassette, respectively, using allelic exchange. All mutant strains were selected using LB media supplemented with 50 µg/ml kanamycin.

### Mouse Infection Assays and Enrofloxacin Treatment

Six to eight-weeks-old female C57BL/6 IL-10^−/−^ mice were infected with 1 × 10^6^ CFU of *S*. Typhimurium 14028 WT or mutant strains ΔTTSS-1, ΔTTSS-2, and ΔSPI-2 contained in 200 µl of sterile saline solution (PBS) by intragastric gavage (i.g.). At 7 days post-infection (days p.i.), mice were treated with 2 mg/ml enrofloxacin (Centrovet Ltda, Santiago, Chile) in drinking water for 21 days. After enrofloxacin treatment, mice received regular drinking water for 14 days until the end of the experiment (42 days p.i.). For C57BL/6 WT mice infection, 1 × 10^5^ CFU of *S*. Typhimurium were administered by i.g. and enrofloxacin treatment was started at day 4 p.i. After 21 days p.i., mice received regular drinking water until the end of the experiment (39 days p.i.). In the acute colitis experimental model, 2% of DSS was added to drinking water from day 38 to day 44 p.i. Percentage of survival, weight variations, and clinical symptoms were evaluated until the end of the experiments and classified according to specific clinical scores.

### Induction of Acute Colitis

Colitis was induced in infected and uninfected mice by administration of drinking water supplemented with 2% DSS (molecular weight 36–50 kDa, Biomedicals, OH, USA) for 7 days. Clinical symptoms were evaluated daily by scoring the body weight loss, changes in stool consistency, and presence of blood in feces. The colitis score was determined as explained in Table S1 in Supplementary Material, according to a protocol modified from Chassaing et al. (32). At 7 days post DSS-colitis induction, mice were euthanized, and ileum and colon segments were stored for histological analyses.

### Hematoxylin–Eosin Staining and Histopathology Analyses

Different portions of the intestine (ileum, ascending colon, transverse colon, descending colon, and cecum) were recovered to assess histopathological damage. Tissue portions were fixed in 4% paraformaldehyde, included in paraffin, cut into micro-sections (3–4 µm thickness), mounted on glass slides, and finally stained with hematoxylin (Harris hematoxylin, Thermo) and eosin Y solution (0.5% aqueous, Merk). Histopathological analyses were performed by a blinded observer, considering different parameters of inflammation, as explained in Table S2 in Supplementary Material. Images were obtained in a Zeiss microscope at the Advance Microscopy Unit of the School of Biological Sciences, Pontificia Universidad Católica de Chile.

### Bacterial Load Quantification

To evaluate bacterial loads in the spleen, liver, and mesenteric lymph nodes (mLNs), samples of the different organs were homogenized, serially diluted in sterile PBS, and seeded onto LB, MacConkey, or *Salmonella-Shigella* agar. Plates were supplemented with kanamycin (50 μg/ml) when mutant strains TTSS-1 or TTSS-2 were tested.

### Enrichment Protocol

*Salmonella enterica* serovar Typhimurium isolation from feces was perform using ISO 6579:2002(E) adapted protocol. Briefly, feces were homogenized, inoculated on 5 ml of APT broth (Difco BD) supplemented with 20 µg/ml of novobiocin (Sigma) and incubated for 24 ± 2 h 37°C. Then, positive samples were cultured on modified semi-solid Rappaport Vassiliadis Agar (MSRV, Oxoid) supplemented with novobiocine (33) and incubated at 41.5 ± 1°C between 24 and 48 h. Samples with white diffuse growing were culture on Xylose Lysine Deoxycholate Agar (XLD, Medium, Difco, BD) 37 ± 1°C for 24 ± 3 h. To confirm that the colonies obtained were *Salmonella*, a PCR to detect the *invA* gene was performed.

### Immunization Assays

To prepare vaccine doses, ΔSPI-2 mutant strain was grown in liquid LB medium with agitation at 37°C until OD_600_ equal to 0.6 was reached. For immunization, IL-10^−/−^ mice received 1 × 10^6^ CFUs of ΔSPI-2 strains, contained in 200 µl of sterile PBS, by intragastric administration. At day 14 post-immunization, mice received a boost under the same conditions described before. At day 21 post-immunization, mice were challenged intragastrically with 1 × 10^6^ CFU of *S*. Typhimurium WT. At 7-days p.i., mice were treated with enrofloxacin (2 mg/ml) in drinking water for 21 days. Percentage of survival, weight loss, and bacterial loads were evaluated until day 42 p.i.

### Anti-*S*. Typhimurium Antibodies Detection in Serum

The detection of anti-*S*. Typhimurium antibodies in serum was determined by enzyme-linked immunosorbent assay (ELISA). Each wells of the ELISA plate coated with 50 µl of a solution of heat killed *S*. Typhimurium grown at OD_600_ 0.5–0.6 and incubated overnight at 4°C. Then, wells were blocked with bovine serum albumin 10% and incubated overnight at 4°C. After three washes, serum of immunized/infected and unimmunized/infected animals obtained at 63 days post-immunization was added to each well in different dilution (1/8; 1/16; 1/32; 1/64; 1/128) and incubated overnight at 4°C. The Donkey anti-mouse IgG HRP (1:1,000) or Goat anti-mouse IgA secondary antibody were added after washing and incubated at room temperature for 2 h. 50 µl of TMB was added and incubated for 5 min in darkness. The reaction was stopped by adding 50 µl of 2N H_2_SO_4_. The absorbance of the plates was measured at 450 nm using an ELISA plate reader. As a positive control, 50 µl of the commercial antibody anti-*S*. Typhimurium (clone 1E6, ab8274, abcam) 1:100 was included in the assay.

### Statistical Analysis

Statistical analyses were performed using Prism v7 (GraphPad Software, San Diego, CA, USA). Multiple comparisons two one-way ANOVA with Bonferroni post-test were used to assess whether the means of more than two groups change significantly with respect a second variable. Body weight changes were analyzed using two-way ANOVA with Dunnetts post-test. Unpaired Mann–Whitney test were used to assess whether the means of two groups differed significantly. Two-way ANOVA analysis was used in some experiment with Kruskal–Wallis post-test.

## Results

### Infection Increases *S*. Typhimurium the Susceptibility of Mice to Develop Intestinal Inflammation due to Acute Chemical Damage

**Figure 1:**
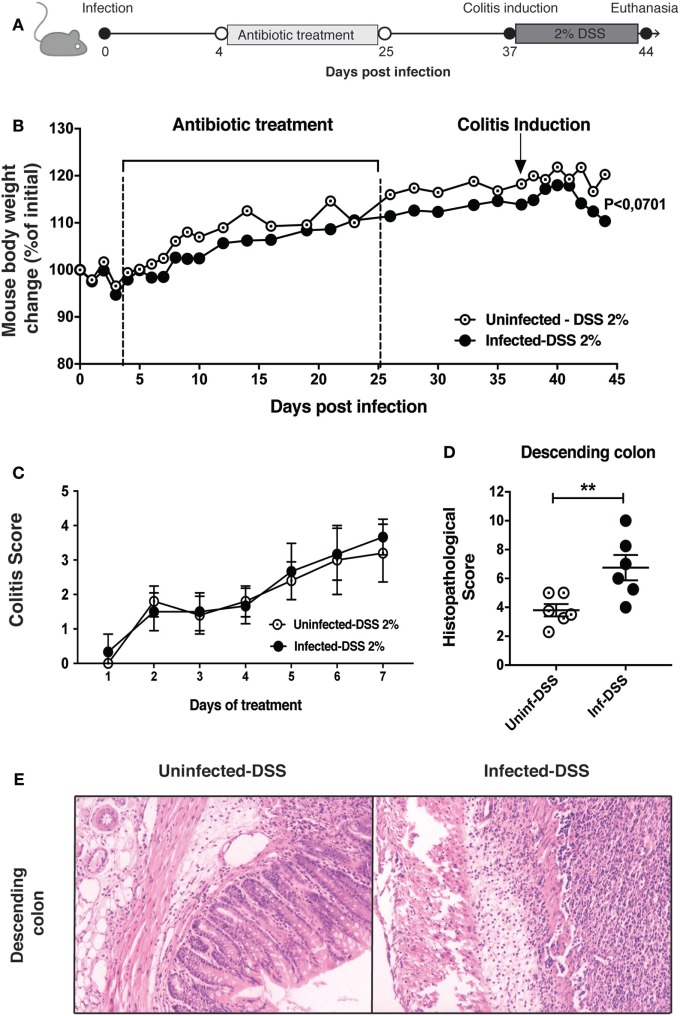
Intestinal inflammation due to colitis induction by dextran sulfate sodium (DSS) 2% is increased by previous *Salmonella enterica* serovar Typhimurium (*S*. Typhimurium) infection. **(A)** C57BL/6 mice were orally infected with 1 × 10^5^ CFU of *S*. Typhimurium wild type (WT). Four days post-infection (days p.i.) mice were orally treated with enrofloxacin (2 mg/ml) for 3 weeks, left untreated for 12 days, and treated orally with DSS 2% for 7 days. **(B)** Body weight of uninfected and previously infected C57BL/6 mice were recorded on a daily basis for 44 days. **(C)** Clinical colitis score recorded during the period of colitis induction by DSS 2%, between 37 and 44 days p.i. **(D)** Average histopathology score of descending colon, performed for three mice per group. **(E)** Representative images of descending colon at day 44 p.i. between uninfected-DSS and infected-DSS treated mice (4 μm descending colon section stained with H&E and observed in optical microscope at 10× magnification). Data shown are mean ± SEM of one experiment with six mice per group. Body weight changes were analyzed using two-way ANOVA with Bonferroni post-test. *P* < 0.07 between uninfected-DSS 2% and infected-DSS 2% treated mice at 44 days p.i. Histopathology score was analyzed by Mann–Whitney *U*-test. Data shown are mean ± SEM *P < 0.05.

To evaluate whether a previous infection with *S*. Typhimurium could promote an increase in intestinal inflammation produced by the administration of DSS 2%, we performed an intragastric infection of C57BL/6 mice with 1 × 10^5^ CFU of virulent *S*. Typhimurium. After 4 days post-infection (days p.i.), mice were treated with enrofloxacin for 21 days to clear bacterial infection (Figure 1A). Then, after 39 days p.i., DSS 2% was administered to mice in drinking water for 7 days. As shown in the Figure 1B, mice infected with *S*. Typhimurium showed a slightly increase in weight loss in the last three days of the treatment with DSS, as compared to control mice. In addition, a colitis score that consider the presence of occult blood and stool aspect (Table S1 in Supplementary Material) was evaluated during the DSS treatment, but it did not show differences between the two groups (Figure 1C). However, at the end of experiment (44 days p.i.), we observed that mice infected with *S*. Typhimurium and treated with DSS 2% showed significantly increased inflammation in descending colon, as compared to uninfected mice that were equally treated with DSS 2% (Figures 1D,E). No pathological features of disease were observed in any other intestinal portions evaluated (Figure S1 in Supplementary Material). These results suggest that WT *S*. Typhimurium leads to an increased intestinal inflammation only in the descending colon, which could be associated to the pathology generated by the administration of DSS.

### *S*. Typhimurium Infection Increases the Susceptibility of IL-10^−/−^ Mice to Develop Spontaneous Intestinal Inflammation in an SPI-2-Dependent Manner

**Figure 2:**
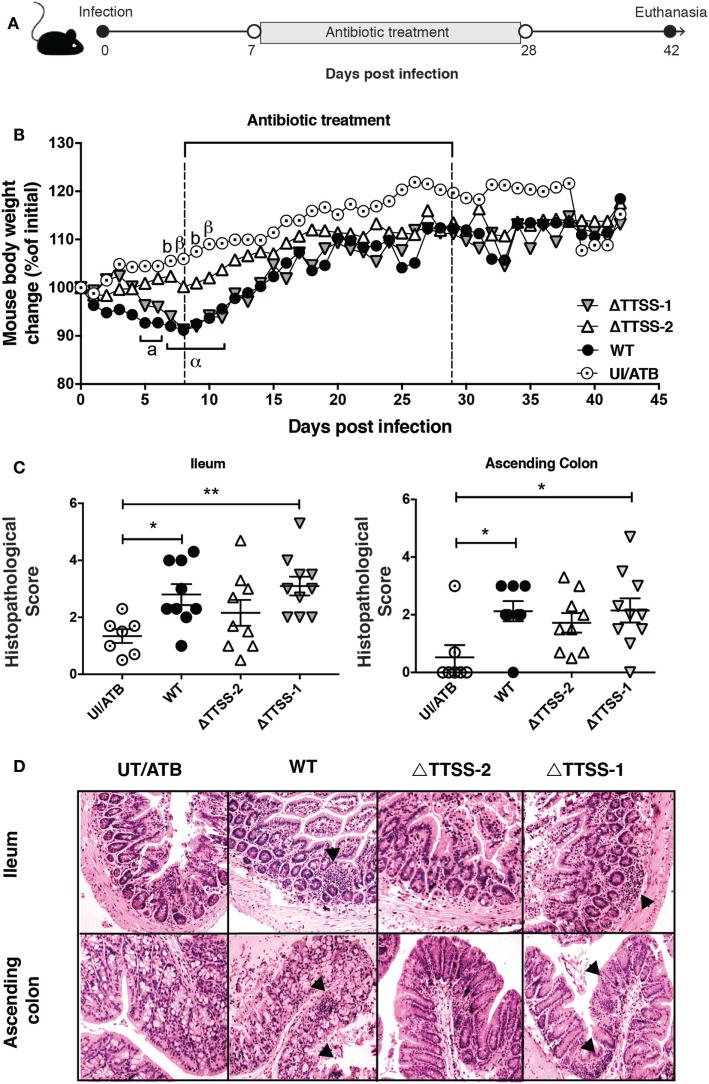
Previous *Salmonella enterica* serovar Typhimurium (*S*. Typhimurium) infection increase the susceptibility of spontaneous intestinal inflammation of interleukin (IL)-10^−/−^ mice. **(A)** IL-10^−/−^ mice were orally infected with 1 × 10^6^ CFU of *S*. Typhimurium (STM) wild type (WT), ΔTTSS-1, or ΔTTSS-2. Seven days post-infection (days p.i.) mice were orally treated with enrofloxacin (2 mg/ml) for 3 weeks, left untreated for 14 days and euthanized to evaluate spontaneous intestinal inflammation. **(B)** Body weight were recorded on a daily basis for 42 days after infection. **(C)** Average histopathology score for ileum and ascending colon of 6–10 IL-10^−/−^ mice included in each group. **(D)** Representative images of on the intestines of IL-10^−/−^ mice infected with *S*. Typhimurium WT, ΔTTSS-1, or ΔTTSS-2 (4 µm ileum and ascending colon section stained with H&E and observed in optical microscope at 10× magnification). Body weight changes were analyzed using two-way ANOVA with Dunnetts post-test. Mice infected with WT (a, α) and TTSS-1 (b, β) were compared with mice uninfected and treated with antibiotics (UI/ATB). Data shown are mean ± SEM of three independent experiment with 8 mice uninfected; 10 mice infected with WT strain; 10 mice infected with ΔTTSS-1 strain; and 9 mice infected with ΔTTSS-2 strain. Statistical significance was indicated by letters: a, b: *P < 0.05; α, β: **P < 0.01. Histopathology score was analyzed by one-way ANOVA with Kruskal–Wallis post-test. Data shown are mean ± SEM *P < 0.05; compared with uninfected mice. Tissue damage and inflammatory infiltration are indicated (arrow).

To evaluate colitis induction in another IBD model, IL-10^−/−^ mice were intragastrically infected with 1 × 10^6^ CFUs of *S*. Typhimurium. After 7 days of infection, mice were treated with enrofloxacin and monitored up to day 42 days p.i. (Figure 2A). For IL-10^−/−^ mice, we used a dose and an infection time that was higher than the dose and time chosen for WT mice, because the former mice are more resistant to *S*. Typhimurium infection (34). As shown in Figure 2B, infected IL-10^−/−^ mice experienced significant weight loss from 1 to 10 days p.i. (showing approximately a 10% weight reduction) and started to recover their weight until the end of the experiment, which correlated with enrofloxacin treatment. At the end of the experiment (42 days p.i.), the intestine was evaluated for spontaneous inflammation and, as observed in Figure 2C, IL-10^−/−^ mice previously infected with virulent *S*. Typhimurium showed an increased inflammation in the ileum and ascending colon, as compared to control uninfected mice.

**Figure 3:**
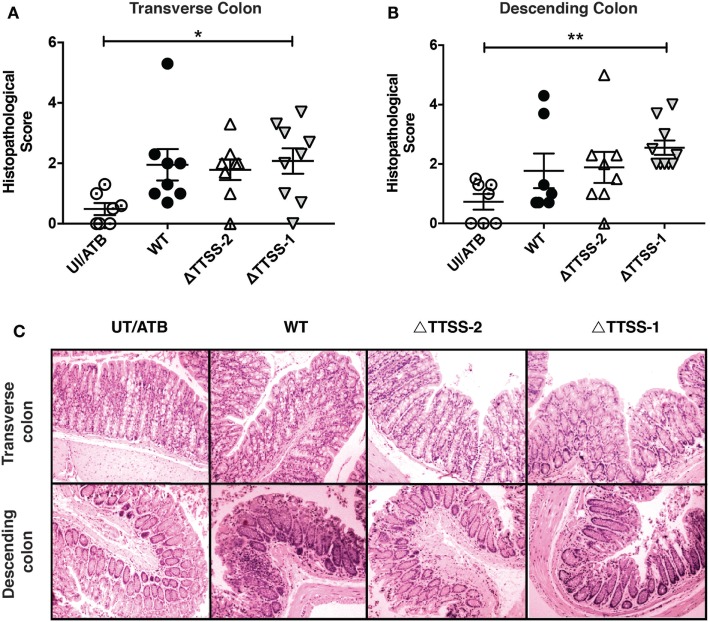
Previous *Salmonella enterica* serovar Typhimurium (*S*. Typhimurium) infection increases the susceptibility of spontaneous inflammation of colon of interleukin (IL)-10^−/−^ mice in a 2-encoded type three secretion system (TTSS-2)-dependent fashion. Average histopathology score for **(A)** transverse colon and **(B)** descending colon of 6–10 IL-10^−/−^ mice included in each group. **(C)** Representative images of the intestines of IL-10^−/−^ mice infected with *S*. Typhimurium wild type (WT), ΔTTSS-1, or ΔTTSS-2 (4 µm transverse and descending colon sections stained with H&E and observed in optical microscope at 10× magnification). Data shown are mean ± SEM of three independent experiment with 8 mice uninfected; 10 mice infected with WT strain; 10 mice infected with ΔTTSS-1 strain; and 9 mice infected with ΔTTSS-2 strain. Histopathology score was analyzed by one-way ANOVA with Kruskal–Wallis post-test. Data shown are mean ± SEM *P < 0.05; compared with uninfected mice. Tissue damage and inflammatory infiltration are indicated (arrow).

Next, we evaluated whether the inflammation of a specific intestinal portion observed in IL-10^−/−^ mice infected with virulent *S*. Typhimurium depended of specific virulence genes of the bacterium. To address this aim, mice were infected with *S*. Typhimurium strains lacking functional TTSS-1 or TTSS-2 and an experiment similar to the one described before was performed (Figure 2A). As shown in Figure 2B, mice infected with the ΔTTSS-1 strain also showed a significant weight loss as compared to uninfected mice, while mice infected with the ΔTTSS-2 strain showed no significant weight loss. Furthermore, a similar pattern of inflammation in ileum and ascendant colon was observed in mice infected with the ΔTTSS-1 strain, while none of the mice infected with the ΔTTSS-2 strain showed significant intestinal inflammation (Figures 2C,D). Intriguingly, mice infected with the ΔTTSS-1 showed increased inflammation also in transverse (Figure 3A) and descending colon (Figure 3B), as compared to mice infected with WT *S*. Typhimurium or control mice (Figure 3C). These results suggest that permanent inflammation in IL-10^−/−^ mice due to *S*. Typhimurium infection depends of the bacterial ability to invade and survive within phagocytic cells, rather than its capacity to invade intestinal epithelium and cause acute infection.

### The Increased Susceptibility of Mice to Develop Intestinal Inflammation due to Previous *S*. Typhimurium Infection Is Associated to Persistent Bacteria in Tissues

**Figure 4:**
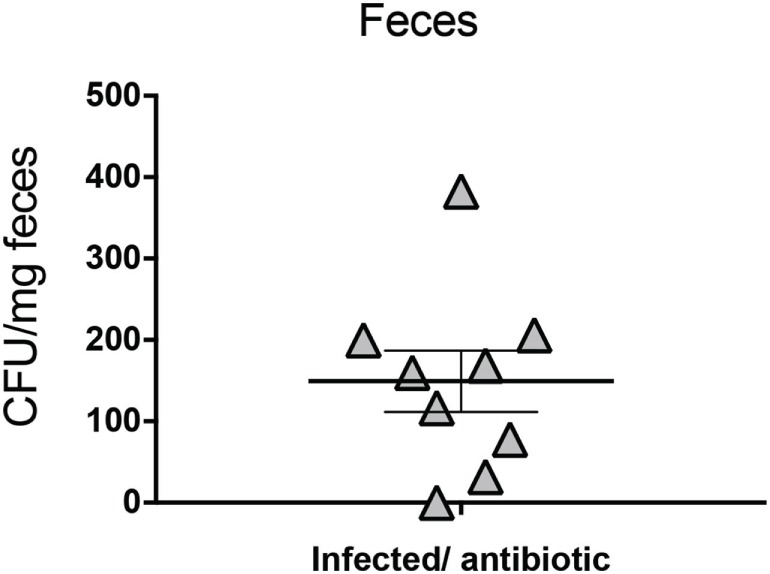
*Salmonella enterica* serovar Typhimurium (*S*. Typhimurium) is excreted in feces after 39 days of infection in wild type (WT) mice, despite enrofloxacin treatment. C57BL/6 WT mice were infected with 1 × 10^5^ CFU of *S*. Typhimurium WT. Four days post-infection (days p.i.) mice were orally treated with enrofloxacin (2 mg/ml) for 3 weeks, and then left untreated for 14 days. Bacterial loads were quantified at 39 days post-infection by seeding feces into *Salmonella-Shigella* agar, as described in Section “Materials and Methods.” Data shown include two separated experiment, nine mice in total.

To determine whether the increased intestinal inflammation in both WT and IL-10^−/−^ mice was associated to a persistent *S*. Typhimurium infection, bacterial loads were evaluated at 39 and 42 days p.i., respectively, in liver, spleen, and mLNs. In both mice strains infected with *S*. Typhimurium WT, the bacteria were detected in the spleen and liver in 20–50% of the infected mice, even after a treatment with enrofloxacin that lasted for 3 weeks (Table 1). As shown in Table 1, there was no difference in the histopathology score in mice that had persistent bacteria in internal organs as compared to those with no persistent bacteria. However, we observed that at 39 days p.i., eight out of nine infected WT mice had significant amounts of *S*. Typhimurium in the feces (Figure 4), although persistent bacteria were not detected in Peyer’s Patches or cecum content (data not shown). Furthermore, we evaluated the effectiveness of the enrofloxacin treatment by enrichment protocol, which confirmed the depletion of the intestinal microbiota during the enrofloxacin treatment, as well as its recovery at the end of the treatment (not shown). These results suggest that most of the mice infected and treated with antibiotics showed a relapsing infection after antibiotic withdrawn, although we were not able to detect the persistent bacteria in deep tissues in all of the mice included in the study. In IL-10^−/−^ mice infected with the ΔTTSS-1 strain, we found that only one of the mice included in all the experiments showed detectable bacterial loads in spleen, liver, and mLNs, while none of the mice infected with the ΔTTSS-2 strain showed bacteria in internal organs (Table 1). These results suggest that the intestinal inflammation induced by *S*. Typhimurium in IL-10^−/−^ mice could depend on the capacity of the bacterium to cause a persistent infection in the host.

**Table 1:** Relationship between presence of persistent *S*. Typhimurium isolated from internal organs vs colonic histological score of mice intragastrically infected and treated for 3 weeks with enrofloxacin.

Experimental group	Days post-infection	Total mice	% of persistent mice	Colon score Persistent bacteria	Colon score No persistent bacteria
C57BL/6 + STM WT	39	6	50	2.3	1.9
IL-10^−/−^ + STM WT	42	9	33.3	1.9	1.5
IL-10^−/−^ + STM ΔTTSS-1	42	10	10	1.33	2.3
IL-10^−/−^ + STM ΔTTSS-2	42	9	0	0	1.7
IL-10^−/−^ unimmunized	63	7	57.1	1.9	1
IL-10^−/−^ immunized	63	8	50	0.4	0.5

*WT or IL-10^−/−^ mice were intragastrically infected with 1 × 10^5^ or 1 × 10^6^ CFU of *S*. Typhimurium, respectively and bacterial loads were quantified at the end of the experiments (39, 42, or 63 days post-infection, respectively) in spleen, liver, and mLNs. The homogenized tissues were cultured into MacConkey agar, as described in Section “Materials and Methods.” The histopathological score of colon (ascending, transverse, and descending colon) did not show differences with respect to non-persistent mice*.

*S. Typhimurium, Salmonella enterica serovar Typhimurium; WT, wild type; TTSS, type three secretion system; mLNs, mesenteric lymph nodes; IL, interleukin*.

### Oral Immunization With an Attenuated Live Vaccine Reduces Intestinal Inflammation, but It Did Not Prevent Persistent Infection in *S*. Typhimurium-Infected IL-10^−/−^ Mice

**Figure 5:**
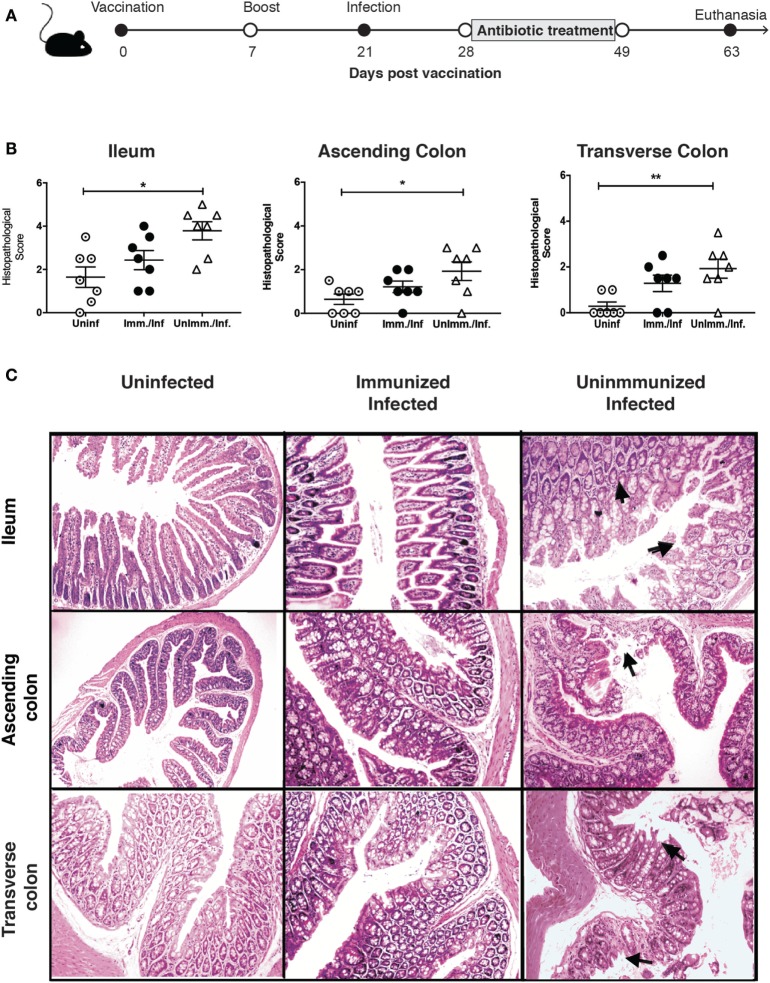
Immunization reduces the increased susceptibility of interleukin (IL)-10^−/−^ for intestinal inflammation due to previous *Salmonella enterica* serovar Typhimurium (*S*. Typhimurium) infection. **(A)** IL-10^−/−^ mice were immunized with 1 × 10^6^ CFU of attenuated *S*. Typhimurium strain ΔSPI-2 at day 0 and received a boost at day 14. Subsequently, mice were challenged with virulent *S*. Typhimurium on day 21. Seven days post-challenge, mice were orally treated with enrofloxacin (2 mg/ml) for 3 weeks, left untreated for 14 days and euthanized at 63 days post-immunization to evaluate spontaneous intestinal inflammation. **(B)** Average histopathology score for ileum, ascending, and transverse colon of seven IL-10^−/−^ mice included in each group. **(C)** Representative images of the intestines of IL-10^−/−^ mice, unimmunized-uninfected mice, unimmunized-infected and immunized-infected mice (4 µm ileum and ascending colon section stained with H&E and observed in optical microscope at 10× magnification). Arrows indicate inflammatory cellular infiltration and erosion. Histological score analyzed by one-way ANOVA with Kruskal–Wallis post-test. Data shown are mean ± SEM of two independent experiments with three to four mice per group. *P < 0.05, compared uninfected with immunized-infected or unimmunized-infected mice.

The results obtained in the previous experiments led us to hypothesize that immunization of IL-10^−/−^ mice with a live attenuated strain of *S*. Typhimurium could be beneficial to prevent bacterial persistence and intestinal inflammation. To approach this hypothesis, IL-10^−/−^ mice were intragastrically immunized with 1 × 10^6^ CFUs of the ΔSPI-2 strain. At day 14, a second oral dose of the same strain was administered to these mice. Mice were then challenged at day 21 post-immunization with virulent *S*. Typhimurium, as described above, to evaluate whether immunization reduced their susceptibility to develop spontaneous intestinal inflammation, as well as bacterial persistence (Figure 5A). To corroborate whether the immunization strategy induced an immune response against *Salmonella*, we examined the anti-*Salmonella* antibodies at 63 days post-immunization in the immunized mice and detected elevated IgG and IgA levels (Figure S2 in Supplementary Material).

In agreement with our previous results, unimmunized and infected IL-10^−/−^ mice showed a significantly higher level of spontaneous inflammation in ileum, ascending colon, and transverse colon, as compared to unimmunized and uninfected IL-10^−/−^ mice (Figure 5B). Importantly, we observed that IL-10^−/−^ mice that were immunized with the ΔSPI-2 strain previous to *S*. Typhimurium infection showed a mild reduction in the susceptibility to develop spontaneous intestinal inflammation in ileum, ascending, and transverse colon (Figure 5B). Despite the histopathological score obtained for immunized and infected mice were not significantly different to the scores obtained for uninfected mice, differences were not found either between the scores obtained for immunized and infected mice and unimmunized and infected mice (Figures 5B,C; Figure S3 in Supplementary Material). These results suggest that immunization could reduce but not prevent the inflammation. Furthermore, bacterial loads in spleens and livers were equivalent in immunized and unimmunized infected mice (Table 1). Importantly, the bacteria found in the internal tissues of immunized mice were the virulent WT *S*. Typhimurium strain used for the challenge, not the vaccine strain, because immunized mice did not show bacterial loads in any tissue previous *S*. Typhimurium WT challenge (data not shown). These results suggest that the vaccine and immunization scheme used here promoted a reduction in the susceptibility of IL-10^−/−^ mice to develop spontaneous inflammation, but it did not prevent *S*. Typhimurium persistence.

## Discussion

Inflammatory bowel diseases are caused by an abnormal immune response in genetic susceptible host, which results in inflammation and intestinal tissue damage. Although the etiology of this disease is poorly understood, it is widely accepted that an inappropriate immune response toward commensal bacteria is a crucial part of the pathogenesis (35). Interestingly, enteric infections caused by pathogens, such as *Salmonella* and *Campylobacter*, have been associated with the onset and development of IBD in humans (13, 23, 36). However, experimental assays to determine whether previous *S*. Typhimurium infection plays a role in the development of IBD in susceptible hosts has not been done yet. Here, we demonstrate that mice previously infected by *S*. Typhimurium generate higher intestinal inflammation in two different mouse models that resemble IBD features.

Although no differences in weight loss or clinical scores were observed between uninfected and previously infected mice during the colitis induction in DSS administration model, mice that were previously infected by *S*. Typhimurium showed an increased inflammation of descending colon after DSS treatment. It is important to note that ascending and transverse colon are the most exposed sites to *Salmonella* infection, as they are closer to the terminal ileum where such infections mainly occur. Importantly, previous *S*. Typhimurium infection also resulted in a significantly enhanced spontaneous inflammation of IL-10^−/−^ mice, which showed higher histopathological score in ileum and ascending colon, as compared to mice that were not previously infected by the bacterium. The exacerbation of the inflammation in these two mouse model of IBD might be due to the permanent consequences of the *S*. Typhimurium infection in the intestine. For instance, previous reports show that recurrent *S*. Typhimurium infection induce the expression of NEU3 and, in consequence, decrease the levels of intestinal alkaline phosphatase in this tissue. The reduction of the alkaline phosphatase affects the levels of intestinal LPS and induces an inflammatory response in mice (37).

Importantly, we observed persistent *S*. Typhimurium infection, mainly in liver and spleen of IL-10^−/−^ and WT mice treated with enrofloxacin after infection. This observation is in agreement with the mouse typhoid model, where it has been described that *Salmonella* persists in biofilms on gallstones (38, 39), as well as in liver, spleen, and mLNs, where the bacteria persist within macrophages (40, 41). Our data are also in agreement with several recent reports showing that antibiotic treatment promotes the selection of “persisters,” which are clones of *Salmonella* that undergo metabolic and genetic modifications during infection of mice treated with antibiotics. These persisters become insensitive to the action of antibiotics and are not able to replicate and reside intracellularly (41–44). Supporting this idea, a recent report has shown that an acetyltransferase toxin denominated TacT, which blocks protein synthesis by affecting the function of tRNA molecules, is required by *S*. Typhimurium to become antibiotic insensitive and persist in the infected tissues of mice (45). Importantly, it has been demonstrated that in the absence of antibiotics, persisters can resume active growth from infected tissues and account for relapsing infections (43, 45). These studies suggest that the reservoir cell for these non-replicating bacteria are able to protect them from the action of the antibiotics, resulting in cell permanently infected.

In the experimental settings of our study, although we detected persistent *S*. Typhimurium in deep organs in around 50% of both C57BL/6 WT and IL-10^−/−^, we did observe a high percentage of mice that shed bacteria in feces at the end of the experiment, when antibiotic treatment was withdrawn. This observation suggests that the method used to detect persistent *S*. Typhimurium infection is not sensitive enough, which might produce false negatives for tissues where the bacterial load is very low. This imply the necessity of more sensitive techniques for the evaluation of persistent *S*. Typhimurium in deeper organs, such as qRT-PCR. These results suggest that the higher susceptibility to develop intestinal inflammation in mice might be related relapsing episodes of *S*. Typhimurium infection, which could be enhancing a pro-inflammatory environment in intestinal tissue. In support of this hypothesis are the results of the immunization assay performed here. Although immunization with ΔSPI-2 strain was unable to prevent persistence of *S*. Typhimurium in spleen and liver of infected mice, it did induce an IgG and IgA humoral immune response against the WT strain. As a matter of fact, immunized and infected mice showed less inflammation that unimmunized and infected mice, despite the presence of persistent bacteria in deep tissues in both groups. It is possible that specific anti-*Salmonella* immunoglobulin levels in serum and mucosa of immunized mice were efficient in the clearance of relapsing bacteria (46, 47), or might potentially generate a different immune response against these re-emerging bacteria, preventing in turn inflammation of the intestinal tissue.

In the streptomycin mouse model, which is efficiently colonized by *S*. Typhimurium, TTSS-2 mutants invade epithelial cells through TTSS-1 and elicit early inflammatory responses in the intestine, but are incapable of spreading systemically (48). This is in agreement with our results, showing that translocation of *Salmonella* effectors *via* TTSS-2 are required to increase spontaneous intestinal inflammation in IL-10^−/−^ mice, because previous infection of the mice with a *S*. Typhimurium ΔTTSS-2 did not result in increased spontaneous intestinal inflammation in the future. Accordingly, previous infection of IL-10^−/−^ mice with a *S*. Typhimurium ΔTTSS-1, that has a functional TTSS-2, is still able to promote inflammation in the intestine. Thus, it is possible that this capacity of *S*. Typhimurium relies on virulence proteins that allow the bacteria to invade and survive inside phagocytic cells. Therefore, our results suggest that *S*. Typhimurium is able to persist inside host cells for long times and requires virulence proteins encoded by the TTSS-2 to generate chronic inflammation of the host’s tissues.

In summary, in this study, we have shown that *S*. Typhimurium infection leads to higher susceptibility to develop intestinal inflammation in two mouse models that resemble IBD, which could be relate to the ability of the bacteria to cause a persistent/relapsing infection, aided by genes in SPI-2. It is of high concern that the persistence of the bacteria occurs despite enrofloxacin treatment and, according to other recent studies, it could be favored by the use of these drugs. Since the intestinal inflammation caused by *S*. Typhimurium can be in part prevented by immunization, it is important to address whether a similar phenomenon occurs in humans, in order to design new prophylactic alternatives to prevent exposition to microbial triggers, as *S*. Typhimurium, which could accelerate IBD onset or exacerbate this disease in susceptible hosts.

## Ethics Statement

All the experiments using mice were conducted in agreement with the ethical standards and according to the local animal protection law. This study was approved by the Scientific Ethical Committee for Animal and Environment Care of the Pontificia Universidad Católica de Chile and the Scientific Committee for Research Biosafety (Protocol number 160715006). Experiments were conducted in agreement to institutional and international Guidelines for Animal Care.

## Author Contributions

BS, GS, CP, CP-R, DP, and FS-E performed the experiments. CR, AK, MA-L, and SB designed the experiments. BS, GS, and SB analyzed the data. BS, GS, AK, and SB wrote the paper. JT and CP-R performed histopathological analyses.

## Conflict of Interest Statement

The authors declare that the research was conducted in the absence of any commercial or financial relationships that could be construed as a potential conflict of interest.

## Supplementary Material

Figure S1 C57BL/6 wild-type (WT) mice previously infected with *Salmonella typhimurium* have similar intestinal tissue damage in ileum, ascending, and transverse colon as uninfected mice. WT mice were orally infected with 10^5^ CFU of *S. typhimurium* WT. **(A)** Histopathology score was analyzed by one-way ANOVA with Kruskal–Wallis post-test. **(B)** At day 44 p.i. the intestines were removed and embedded in paraffin. 4 μm section of ileum, ascending, and transverse colon section were stained with H&E and observed in optical microscope at 10× magnification. Data show mean ± SEM ns, non-significant; compared with uninfected-treated with 2% DSS.

Figure S2 Immunization of IL-10^−/−^ mice did not prevent increase susceptibility to develop inflammation in descending colon due to previous *S. typhimurium* infection. **(A)** At day 63 post immunization, the intestines were removed and embedded in paraffin. 4 μm sections of descending colon section were stained with H&E and observed in optical microscope at 10× magnification. **(B)** Histological score was analyzed by one-way ANOVA with Kruskal–Wallis post-test. Data show mean ± SEM ns, non-significant; compared uninfected mice with immunized-infected mice or unimmunized-infected mice. Differences between groups immunized-infected and unimmunized-infected were found using ANOVA and Tukey as post-test, **P* < 0.05. Infiltrated and erosion are indicated respectively (arrow).

Figure S3 Immunization of IL-10^−/−^ mice increase antibody immune response against *S. typhimurium* WT. **(A,B)** The measurement of IgA against *S. typhimurium* WT was made in ileum and colon content of IL-10^−/−^ mice immunized with ΔSPI-2 and challenged with *S. typhimurium* WT at day 63 post immunization as described in material and methods. **(C)** The measure of IgG against *S. typhimurium* WT was made in serum of IL-10^−/−^ mice immunized with ΔSPI-2 and challenged with *S. typhimurium* WT at day 63 post immunization as described in material and methods. Data show mean ± SEM compared immunized-infected mice or unimmunized-infected mice. Differences between groups immunized-infected and unimmunized-infected were found using ordinary one-way ANOVA, ***P* < 0.05.

## Abbreviations

*S*. Typhimurium, *Salmonella enterica* serovar Typhimurium; SPI, *Salmonella* pathogenicity island; IBD, inflammatory bowel disease; DSS, dextran sulfate sodium; IL-10, interleukin-10; TTSS, type three secretion system; WT, wild type; p.i., post-infection.
