# Supplementary figures and images for: Persistent Salmonella enterica serovar Typhimurium Infection Increases the Susceptibility of Mice to Develop Intestinal Inflammation
Source: Front Immunol. 2018 May 29;9:1166. doi: 10.3389/fimmu.2018.01166 (PMC5986922; doi:10.3389/fimmu.2018.01166)

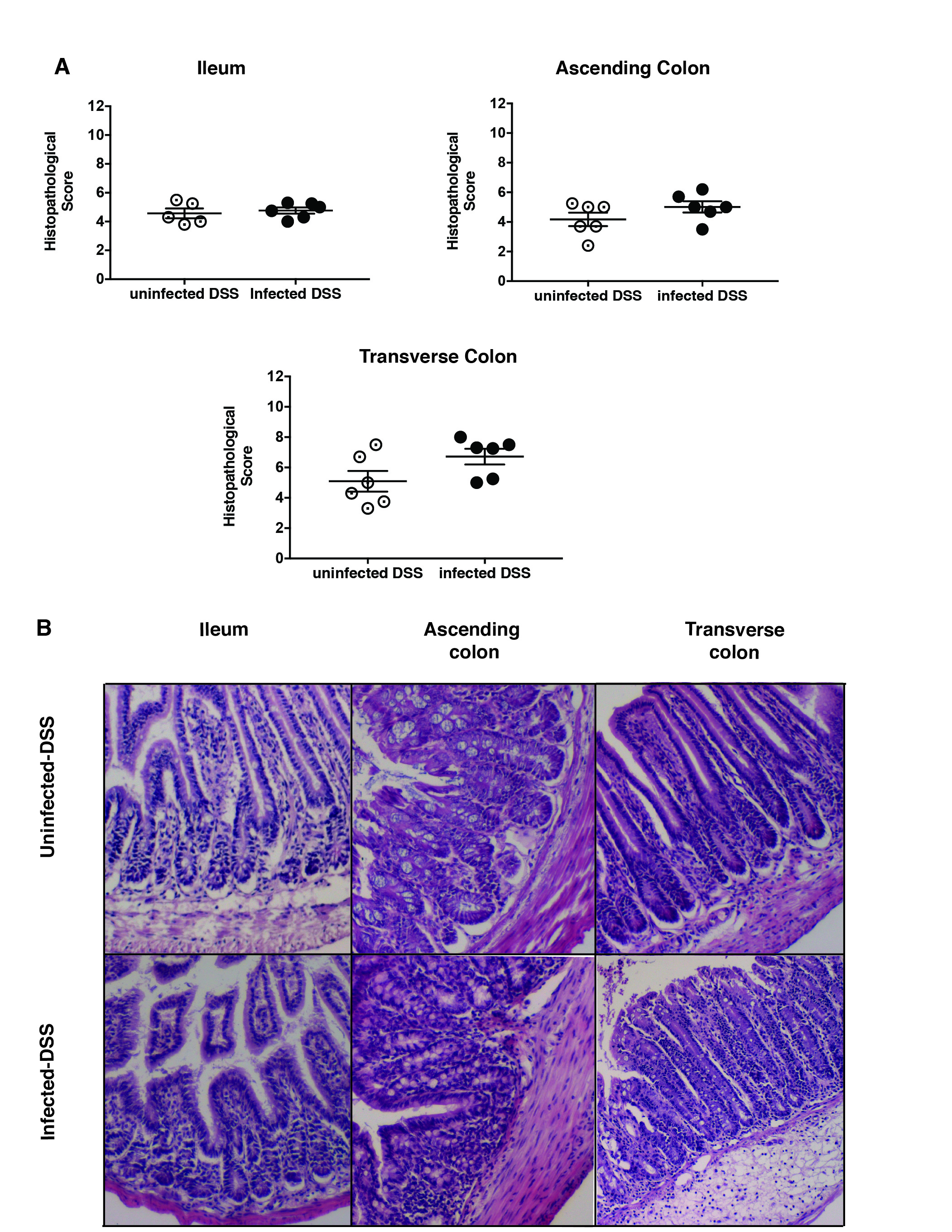

Supplement: Figure S1 — C57BL/6 wild-type (WT) mice previously infected with Salmonella typhimurium have similar intestinal tissue damage in ileum, ascending, and transverse colon as uninfected mice. WT mice were orally infected with 105 CFU of S. typhimurium WT. (A) Histopathology score was analyzed by one-way ANOVA with Kruskal–Wallis post-test. (B) At day 44 p.i. the intestines were removed and embedded in paraffin. 4 μm section of ileum, ascending, and transverse colon section were stained with H&E and observed in optical microscope at 10× magnification. Data show mean ± SEM ns, non-significant; compared with uninfected-treated with 2% DSS. [file Image_1.jpeg]

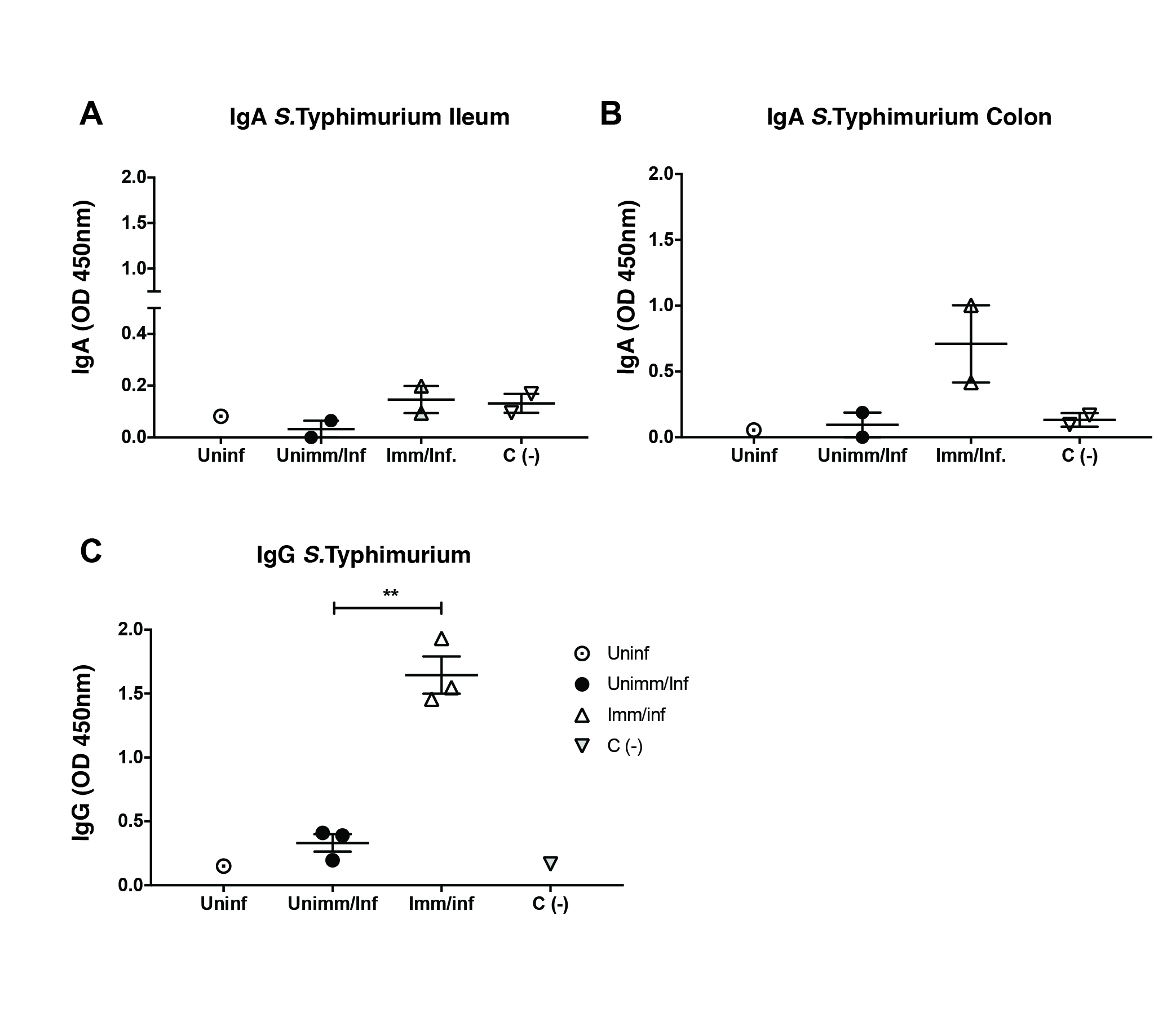

Supplement: Figure S2 — Immunization of IL-10−/− mice did not prevent increase susceptibility to develop inflammation in descending colon due to previous S. typhimurium infection. (A) At day 63 post immunization, the intestines were removed and embedded in paraffin. 4 μm sections of descending colon section were stained with H&E and observed in optical microscope at 10× magnification. (B) Histological score was analyzed by one-way ANOVA with Kruskal–Wallis post-test. Data show mean ± SEM ns, non-significant; compared uninfected mice with immunized-infected mice or unimmunized-infected mice. Differences between groups immunized-infected and unimmunized-infected were found using ANOVA and Tukey as post-test, *P < 0.05. Infiltrated and erosion are indicated respectively (arrow). [file Image_2.tif]

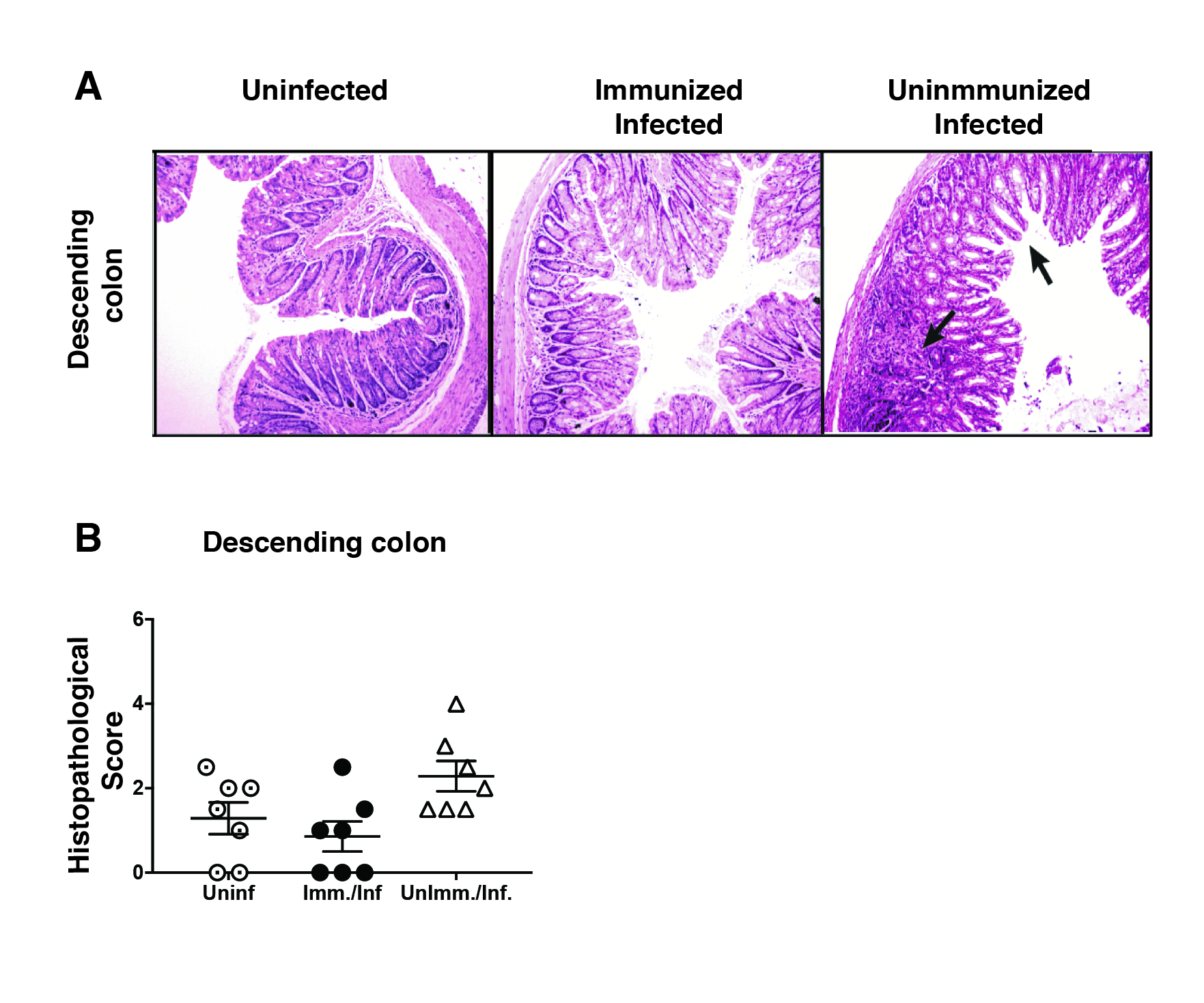

Supplement: Figure S3 — Immunization of IL-10−/− mice increase antibody immune response against S. typhimurium WT. (A,B) The measurement of IgA against S. typhimurium WT was made in ileum and colon content of IL-10−/− mice immunized with ΔSPI-2 and challenged with S. typhimurium WT at day 63 post immunization as described in material and methods. (C) The measure of IgG against S. typhimurium WT was made in serum of IL-10−/− mice immunized with ΔSPI-2 and challenged with S. typhimurium WT at day 63 post immunization as described in material and methods. Data show mean ± SEM compared immunized-infected mice or unimmunized-infected mice. Differences between groups immunized-infected and unimmunized-infected were found using ordinary one-way ANOVA, **P < 0.05. [file Image_3.tif]
