# Supplementary material for: Persistent Salmonella enterica serovar Typhimurium Infection Increases the Susceptibility of Mice to Develop Intestinal Inflammation
Source: Front Immunol. 2018 May 29;9:1166. doi: 10.3389/fimmu.2018.01166 (PMC5986922; doi:10.3389/fimmu.2018.01166)
Supplement: Supplementary file 4 [file Table_1.docx]

**Supplementary Table S1:**

**Clinical score for DSS induce colitis mice model.** We performed two independent score: The observation of stool consistency ranging from 0 to 4, and the occult blood assay considered in the final score as absence (0) or presence (1). The maximum score for the colitis induction by 2% DSS, could be 5 with the sum of both score, however, if the mouse presented tenesmus, this was considered as maximum score due to this process is an extreme inflammation of the intestine.

|  | Score of feces | Occult blood |  |
| --- | --- | --- | --- |
| 0 | Normal and well formed | Negative (No blood detected) |  |
| 1 | Loose stool | Positive + (Traces) |  |
| 2 | Watery stool | Positive ++ (moderated) |  |
| 3 | Watery stools with visible rectal bleeding | Positive +++ (Gross bleeding) |  |
| 4 | Tenesmus (straining at stool) | - |  |
