# Supplementary material for: Persistent Salmonella enterica serovar Typhimurium Infection Increases the Susceptibility of Mice to Develop Intestinal Inflammation
Source: Front Immunol. 2018 May 29;9:1166. doi: 10.3389/fimmu.2018.01166 (PMC5986922; doi:10.3389/fimmu.2018.01166)
Supplement: Supplementary file 5 [file Table_2.docx]

**Supplementary Table S2**:

**Histopathological scoring for intestinal tissue from WT and IL-10^-/-^ mice.** The degree of inflammation and related histologic changes were evaluated using a validated scoring system; score 0: to normal tissue and score 11: severe inflammation and damage.

| Pathological Characteristics | Score (in parentheses) |
| --- | --- |
| Distortion of Architecture | Presence (1)  Absence (0) |
| Epithelial Dedifferentiation | Presence (1)  Absence (0) |
| Cryptitis | Presence (1)  Absence (0) |
| Microabscess | Presence (1)  Absence (0) |
| Erosion | Presence (1)  Absence (0) |
| Ulcer | Presence (1)  Absence (0) |
| Granuloma | Presence (1)  Absence (0) |
| Inflammatory infiltrated | Presence (1)  Absence (0) |
| Gravity Infiltrated | Slight (1)  Mild (2)  Severe (3) |
| Localization infiltrated | Mucosa (0)  Submucosa (0)  Lámina Propria (0)  Muscular (0) |
| Activity index | 0 (healthy) –  11 (severe inflammation and damage) |
