# Supplementary material for: Persistent Salmonella enterica serovar Typhimurium Infection Increases the Susceptibility of Mice to Develop Intestinal Inflammation
Source: Front Immunol. 2018 May 29;9:1166. doi: 10.3389/fimmu.2018.01166 (PMC5986922; doi:10.3389/fimmu.2018.01166)
Supplement: Supplementary file 6 [file Data_Sheet_1.docx]

**Figure S1: C57BL/6 WT mice previously infected with *S.* Typhimurium have similar intestinal tissue damage in ileum, ascending and transverse colon as uninfected mice.** WT mice were orally infected with 10^5^ CFU of *S.* Typhimurium WT. (A) Histopathology score was analyzed by one-way ANOVA with Kruskal Wallis post-test. (B) At day 44 p.i. the intestines were removed and embedded in paraffin. 4um section of Ileum, Ascending and Transverse colon section were stained with H&E and observed in optical microscope at 10X magnification. Data show mean + SEM ns: non-significant; compared with Uninfected-treated with 2% DSS.

**Figure S2:** **Immunization of IL-10^-/-^ mice did not prevent increase susceptibility to develop inflammation in descending colon due to previous *S.* Typhimurium infection.** (A) At day 63 post immunization, the intestines were removed and embedded in paraffin. 4µm sections of descending colon section were stained with H&E and observed in optical microscope at 10X magnification. (B) Histological score was analyzed by one-way ANOVA with Kruskal Wallis post-test. Data show mean with + SEM ns: non-significant; compared Uninfected mice with Immunized-Infected mice or Unimmunized-Infected mice. Differences between groups Immunized-Infected and Unimmunized-Infected were found using ANOVA and Tukey as post-test, **P* < 0.05. Infiltrated and erosion are indicated respectively (arrow).

**Figure S3: Immunization of IL-10^-/-^ mice increase antibody immune response against *S.* Typhimurium WT.** (A-B) The measurement of IgA against *S.* Typhimurium WT was made in ileum and colon content of IL-10^-/-^ mice immunized with ΔSPI-2 and challenged with *S.* Typhimurium WT at day 63 post immunization as described in material and methods. (C) The measure of IgG against *S.* Typhimurium WT was made in serum of IL-10^-/-^ mice immunized with ΔSPI-2 and challenged with *S.* Typhimurium WT at day 63 post immunization as described in material and methods. Data show mean with + SEM compared Immunized-Infected mice or Unimmunized-Infected mice. Differences between groups Immunized-Infected and Unimmunized-Infected were found using ordinary one-way ANOVA, ***P* < 0.005.
